# Prognostic Value of Serum Albumin Level in Critically Ill Patients: Observational Data From Large Intensive Care Unit Databases

**DOI:** 10.3389/fnut.2022.770674

**Published:** 2022-06-13

**Authors:** Xuting Jin, Jiamei Li, Lu Sun, Jingjing Zhang, Ya Gao, Ruohan Li, Jiajia Ren, Yanli Hou, Dan Su, Jiao Liu, Xiaochuang Wang, Dechang Chen, Gang Wang, Christian J. Wiedermann

**Affiliations:** ^1^Department of Critical Care Medicine, The Second Affiliated Hospital of Xi'an Jiaotong University, Xi'an, China; ^2^Department of Ultrasound, The Second Affiliated Hospital of Xi'an Jiaotong University, Xi'an, China; ^3^Department of Cardiology, The Second Affiliated Hospital of Xi'an Jiaotong University, Xi'an, China; ^4^Department of Critical Care Medicine, Ruijin Hospital, Shanghai Jiao Tong University School of Medicine, Shanghai, China; ^5^Institute of Medical Decision Making, Public Health and Health Technology Assessment, UMIT Tirol - University of Health Sciences, Medical Informatics and Technology, Hall in Tyrol, Austria

**Keywords:** serum albumin, mortality, cirrhosis, intensive care unit, database

## Abstract

**Background:**

Decreased serum albumin level (SAL) is associated with adverse clinical outcomes. We designed the present study to further assess the prognostic value of SAL in critically ill patients based on data from large intensive care unit (ICU) databases.

**Methods:**

This retrospective cohort study recruited 18,353 patients from the Medical Information Mart for Intensive Care IV (MIMIC-IV) database. Restricted cubic splines (RCS) were performed to visualize the association of SAL at admission with ICU and hospital mortalities. The prognostic value of SAL was analyzed using logistic regression models and receiver operating characteristic (ROC) curves in overall patients and subgroups.

**Results:**

Restricted cubic splines revealed rapid increasing risks in ICU and hospital mortalities when SAL declined to below 30 g/l. Patients with SAL <30 g/l (n = 6,069) had higher ICU (13.7% vs. 6.4%, *p* < 0.001) and hospital (23.9% vs. 10.7%, *p* < 0.001) mortalities than those with SAL ≥30 g/l. Multivariable logistic regression model revealed that SAL <30 g/l independently correlated with higher risks of both ICU (odds ratio [OR]: 1.20, 95% confidence interval [CI]: 1.07–1.36) and hospital (OR: 1.51, 95% CI: 1.37–1.66) mortalities. However, the association diminished in patients with cirrhosis (OR: 1.16, 95% CI: 0.91–1.49 for ICU mortality; OR: 1.21, 95% CI: 1.00–1.48 for hospital mortality). ROC curves revealed a poor performance of SAL in predicting mortalities, both in overall patients and in those with cirrhosis.

**Conclusions:**

Decreased SAL is associated with increased risk of mortality. However, it possesses low sensitivity and specificity for outcome prediction in critically ill patients, especially in those with cirrhosis.

## Introduction

Critically ill patients were frequently observed with decreased serum albumin levels (SALs) (1, 2), which could result from redistribution of albumin between the vascular and interstitial spaces, nutritional deficiency, increased loss of albumin, and impaired hepatic albumin synthesis (3–5). Besides, administration of intravenous fluids could further decrease SAL through dilution (6). Considering the predominant role of albumin in osmotic pressure maintenance, molecular binding and transporting, anti-oxidation, anti-inflammation, and endothelial stabilization (1, 7, 8), decreased SAL may be a potential predictive indicator for adverse outcomes in critically ill patients.

Previous studies have reported that a low SAL is associated with adverse outcomes in patients with various clinical conditions, such as coronary heart disease, stroke, cancer, sepsis, and chronic obstructive pulmonary disease (6, 9–14). However, the prognostic value of admission SAL for ICU patients has been controversial in previous studies (2, 15–22). Therefore, we carried out the present study to further assess the association of the admission SAL with ICU and hospital mortalities using data from the Medical Information Mart for Intensive Care IV (MIMIC-IV) database and the eICU Collaborative Research Database (eICU-CRD). Both databases provide open access to de-identified large-scale datasets of patients admitted to ICUs, and are available for analyses (23–26).

## Materials and Methods

### Data Source

This retrospective, observational cohort study was based on the data from the MIMIC-IV (version 1.0, updated on March 16, 2021) database, which included 382,278 patients admitted to ICUs at Boston's Beth Israel Deaconess Medical Centre during the period 2008–2019 (27). External validation was performed using the data from the eICU-CRD (version 2.0, published on April 15, 2019), which is a multicenter database including records of 139,367 patients admitted to 335 units at 208 hospitals located throughout the United States between 2014 and 2015 (25). Both databases were developed by the Laboratory for Computational Physiology at Massachusetts Institute of Technology (MIT; Cambridge, MA, USA). As all protected health information from the two databases had been de-identified, the requirement for individual patient consent was waived. All the authors of this manuscript have completed the necessary training, and their requests to access the database were approved.

### Study Population

Patients with serum albumin measurements conducted at the first recorded ICU admission were included in the study. The exclusion criteria were as follows: (1) age <15 years at ICU admission; (2) incomplete data on the covariates or outcomes (Supplementary Figures S1, S2).

### Outcomes

The primary outcome was ICU mortality, defined as death for any reason before ICU discharge. The secondary outcome was hospital mortality, which also included the ICU mortality.

### Data Extraction

The primary independent variable was SAL at admission, which was defined as the first SAL that was measured during the first 24 h of ICU stay. If SAL was not measured during this period, the latest SAL recorded within 24 h prior to ICU admission was adopted instead. We extracted the following covariates for each patient, including the demographic information (age, gender, ethnicity), initial sequential organ failure assessment (SOFA) score (for patients in MIMIC-IV) or Acute Physiology and Chronic Health Evaluation IV (APACHE-IV) score (for patients in eICU-CRD), the use of dialysis and ventilation, comorbidities in accordance with International Classification of Diseases-9th Revision (ICD-9) and ICD-10 codes (diabetes, hypertension, malignant tumor, sepsis, trauma, cirrhosis, hepatic failure, congestive heart failure, respiratory failure, and renal failure), and the discharge status of the patients. In the MIMIC-IV database, the SOFA score was evaluated based on the clinical and laboratory data during the first 24 h after admission (28). Sepsis was diagnosed in accordance with the Sepsis-3 criteria (29). Data were extracted using PostgreSQL program (version 13).

### Statistical Analysis

Continuous variables were presented as the median with interquartile range (IQR) and were compared using the Mann-Whitney U test. Categorical variables were presented as frequencies with percentages and were compared using the Chi-square or Fisher's exact test. SAL was first categorized into 10 groups according to its deciles. The correlations between SAL deciles and mortality rates were measured by Spearman rank sum correlation tests and visualized by locally weighted scatterplot smoothing curves. The relationships between SAL and the odds ratio (OR) of ICU and hospital mortalities were assessed by using restricted cubic splines (RCS) with four knots located in the 5th, 35th, 65th, and 95th percentiles, in accordance with the Harrell Rule (30). Receiver operating characteristic (ROC) curves and areas under curve (AUC) values were used to determine the optimal cut-off value of SAL and assess the prognostic value of SAL and SOFA score. We investigated the association between the SAL, as a continuous or a categorical variable, and mortalities by using univariable and multivariable logistic regression analyses, and reported the ORs and 95% confidence intervals (CIs). After literature review, we used the directed acyclic graphs to confirm confounding factors (31), which were included in the multivariable models. Furthermore, interaction and subgroup analyses were conducted after stratifying the patients based on the confounding factors. All statistical analyses were conducted using R program (version 4.0.3). A two-sided *p*-value <0.05 was considered statistically significant.

## Results

### Patients' Characteristics and SAL Groups

The present study ultimately recruited 18,353 patients from the MIMIC-IV database. Among those patients, 9,563 (52.1%) were older than 65 years, 10,348 (56.4%) were males, and 12,148 (66.2%) were White. The median (IQR) of the SOFA score was 4 (2–7). In total, 1,622 (8.8%) patients died during the ICU stay, and 2,764 (15.1%) patients died during the hospital stay. The median (IQR) of the SAL at admission was 33 (28–38) g/l in this study population (Table 1).

**Table 1:** Demographic and clinical characteristics at baseline for patients from Medical Information Mart for Intensive Care IV database.

	**All (*N* = 18,353)**	**Albumin <30 g/l (*N* = 6,069)**	**Albumin ≥30 g/l (*N* = 12,284)**	***P***
Albumin level, Median (IQR), g/l	33 (28–38)	25 (22–28)	36 (33–40)	<0.001
Age > 65 years, *n* (%)	9,563 (52.1)	3,134 (51.6)	6,429 (52.3)	0.382
Male, *n* (%)	10,348 (56.4)	3,281 (54.1)	7,067 (57.5)	<0.001
**Ethnicity**, ***n*** **(%)**				0.075
White	12,148 (66.2)	3,963 (65.3)	8,185 (66.6)	
Others	6,205 (33.8)	2,106 (34.7)	4,099 (33.4)	
SOFA score, Median (IQR)	4 (2–7)	6 (3–9)	4 (2–6)	<0.001
**Comorbidities**, ***n*** **(%)**
Diabetes	5,167 (28.2)	1,695 (27.9)	3,472 (28.3)	0.647
Hypertension	9,218 (50.2)	2,839 (46.8)	6,379 (51.9)	<0.001
Malignant tumor	2,808 (15.3)	1,400 (23.1)	1,408 (11.5)	<0.001
Sepsis	10,192 (55.5)	4,295 (70.8)	5,897 (48.0)	<0.001
Trauma	2,026 (11.0)	564 (9.3)	1,462 (11.9)	<0.001
Cirrhosis	2,080 (11.3)	1,012 (16.7)	1,068 (8.7)	<0.001
Hepatic failure	618 (3.4)	334 (5.5)	284 (2.3)	<0.001
Congestive heart failure	4,813 (26.2)	1,477 (24.3)	3,336 (27.2)	0.007
Respiratory failure	4,968 (27.1)	2,249 (37.1)	2,719 (22.1)	<0.001
Renal failure	6,832 (37.2)	3,062 (50.5)	3,770 (30.7)	<0.001
**Treatments**, ***n*** **(%)**
Ventilation	13,552 (73.8)	4,780 (78.8)	8,772 (71.4)	<0.001
Dialysis	592 (3.2)	268 (4.4)	324 (2.6)	<0.001
**Outcomes**, ***n*** **(%)**
Hospital death	2,764 (15.1)	1,448 (23.9)	1,316 (10.7)	<0.001
ICU death	1,622 (8.8)	830 (13.7)	792 (6.4)	<0.001

*SOFA, sequential organ failure assessment; IQR, inter-quartile range; ICU, intensive care unit*.

**Figure 1:**
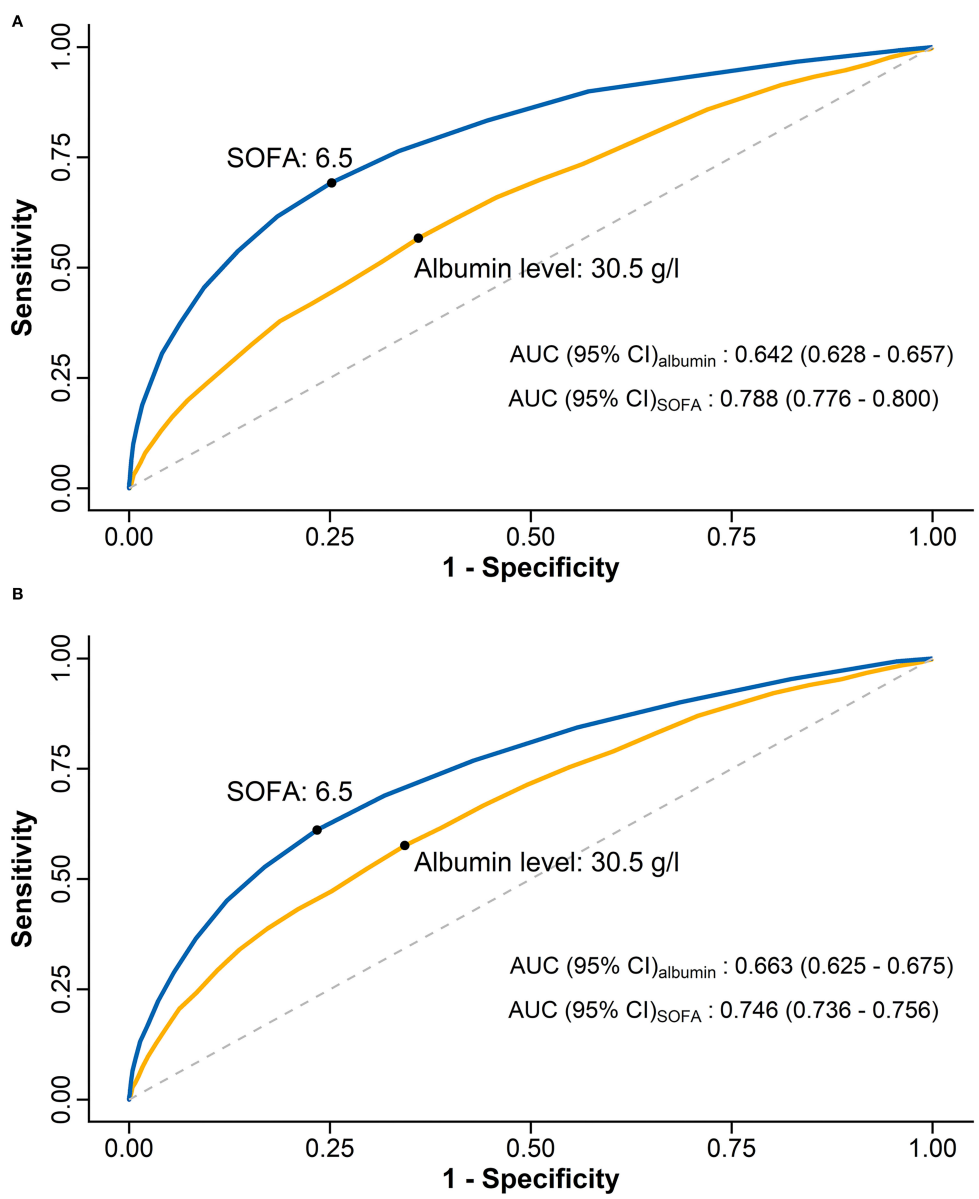
ROC curves and AUC values for admission serum albumin level and SOFA score in predicting ICU **(A)** and hospital **(B)** mortalities. ROC, Receiver operating characteristic; AUC, areas under curve; SOFA score, Sequential Organ Failure Assessment score; ICU, intensive care unit.

The observed ICU (*R* = −0.96, *p* < 0.001) and hospital (*R* = −0.98, *p* < 0.001) mortality rates were negatively correlated with the deciles of SAL (Supplementary Figure S3). The ROC analysis determined that SAL of 30.5 g/l was the optimal threshold which possessed the highest sum of sensitivity and specificity (Figure 1). Based on the literature review and considering clinical practicality, we adopted SAL of 30 g/l as the cut-off value in the present study (1–3). The patients were further dichotomized according to SAL <30 or ≥30 g/l (*n* = 6,069, 33.1%; *n* = 12,284, 66.9%, respectively).

Patients in the group with SAL <30 g/l were less likely to be males (*p* < 0.001); had higher SOFA scores (*p* < 0.001); had higher prevalence rates of malignant tumor (*p* < 0.001), sepsis (*p* < 0.001), cirrhosis (*p* < 0.001), hepatic failure (*p* < 0.001), respiratory failure (*p* < 0.001), and renal failure (*p* < 0.001); and had a higher proportion of ventilation (*p* < 0.001) and dialysis (*p* < 0.001) use. In contrast, the proportion of hypertension (*p* < 0.001), congestive heart failure (*p* = 0.007), and trauma (*p* < 0.001) patients was lower in patients with SAL <30 g/l. Higher ICU and hospital mortalities (13.7 vs. 6.4%, *p* < 0.001; 23.9 vs. 10.7%, *p* < 0.001, respectively) were observed in patients with SAL <30 g/l when compared with those with SAL ≥30 g/l.

### SAL and Mortality Risks

**Figure 2:**
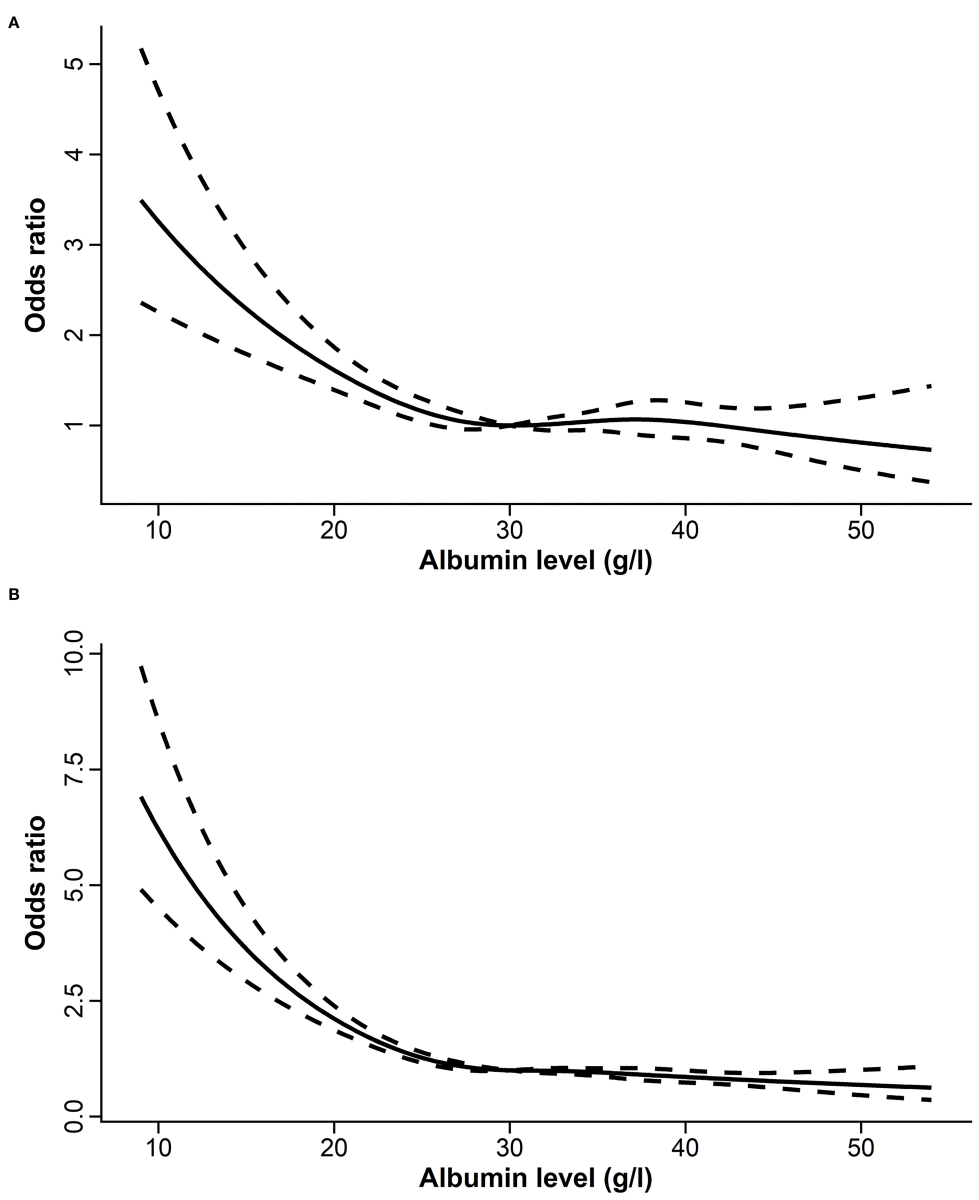
The relationships between serum albumin level and the risk of ICU **(A)** and hospital **(B)** mortalities assessed by restricted cubic splines. ICU, intensive care unit.

Restricted cubic splines revealed rapid increasing risks in ICU and hospital mortalities when SAL declined to below 30 g/l (Figure 2). As a continuous variable, SAL was inversely associated with the risk of ICU and hospital mortalities (OR: 0.98, 95% CI: 0.97–0.99, *p* < 0.001; OR: 0.96, 95% CI: 0.95–0.96, *p* < 0.001, respectively) even after multivariable adjustments. Logistic regression models showed that patients with SAL <30 g/l had higher risks of ICU and hospital mortalities (OR: 2.30, 95% CI: 2.07–2.55, *p* < 0.001; OR: 2.61, 95% CI: 2.41–2.84, *p* < 0.001, respectively) than patients with SAL >30 g/l. After multivariable adjustments, the associations were diminished but still robust (OR: 1.20, 95% CI: 1.07–1.36, *p* = 0.002 for ICU mortality; OR: 1.51, 95% CI: 1.37–1.66, *p* < 0.001, for hospital mortality) (Table 2).

**Table 2:** The association of serum albumin level with mortality risks.

	**ICU mortality**		**Hospital mortality**	
	**OR (95% CI)**	***P***	**OR (95% CI)**	***P***
**MIMIC-IV**
**Albumin level as a continuous variable**
Univariable	0.93 (0.92–0.94)	<0.001	0.92 (0.91–0.92)	<0.001
Adjusted*	0.98 (0.97–0.99)	<0.001	0.96 (0.95–0.96)	<0.001
**Albumin** ** <30 g/L vs. albumin** **≥30g/L (as reference)**
Univariable	2.30 (2.07–2.55)	<0.001	2.61 (2.41–2.84)	<0.001
Adjusted*	1.20 (1.07–1.36)	0.002	1.51 (1.37–1.66)	<0.001
**eICU-CRD**
**Albumin level as a continuous variable**
Univariable	0.91 (0.91–0.91)	<0.001	0.91 (0.90–0.91)	<0.001
Adjusted*	0.97 (0.96–0.97)	<0.001	0.96 (0.95–0.96)	<0.001
**Albumin** ** <30 g/L vs. albumin** **≥30g/L (as reference)**
Univariable	3.16 (2.97–3.36)	<0.001	3.28 (3.12–3.44)	<0.001
Adjusted*	1.43 (1.33–1.54)	<0.001	1.62 (1.52–1.71)	<0.001

^*^Model was adjusted for age, gender, ethnicity, initial SOFA score (for patients from MIMIC-IV database), initial APACHE IV score (for patients from eICU-CRD database), comorbidities including hypertension, diabetes, heart failure, cirrhosis, hepatic disease, respiratory failure, renal failure, cancer, sepsis, trauma, and treatments including ventilation and dialysis.

*ICU, intensive care unit; MIMIC-IV, Medical Information Mart for Intensive Care IV; eICU-CRD, eICU Collaborative Research Database; OR, odds ratio; CI, confidence interval; SOFA, sequential organ failure assessment; APACHE IV, acute physiology and chronic health evaluation IV*.

ROC curves showed an AUC value (95% CI) of 0.642 (0.628–0.657) for SAL in predicting ICU mortality and 0.663 (0.625–0.675) in predicting hospital mortality. Meanwhile, the AUC values (95% CI) for initial SOFA score were 0.778 (0.776–0.800) in predicting ICU mortality and 0.746 (0.736–0.756) in predicting hospital mortality (Figure 1).

### Subgroup Analysis

**Figure 3:**
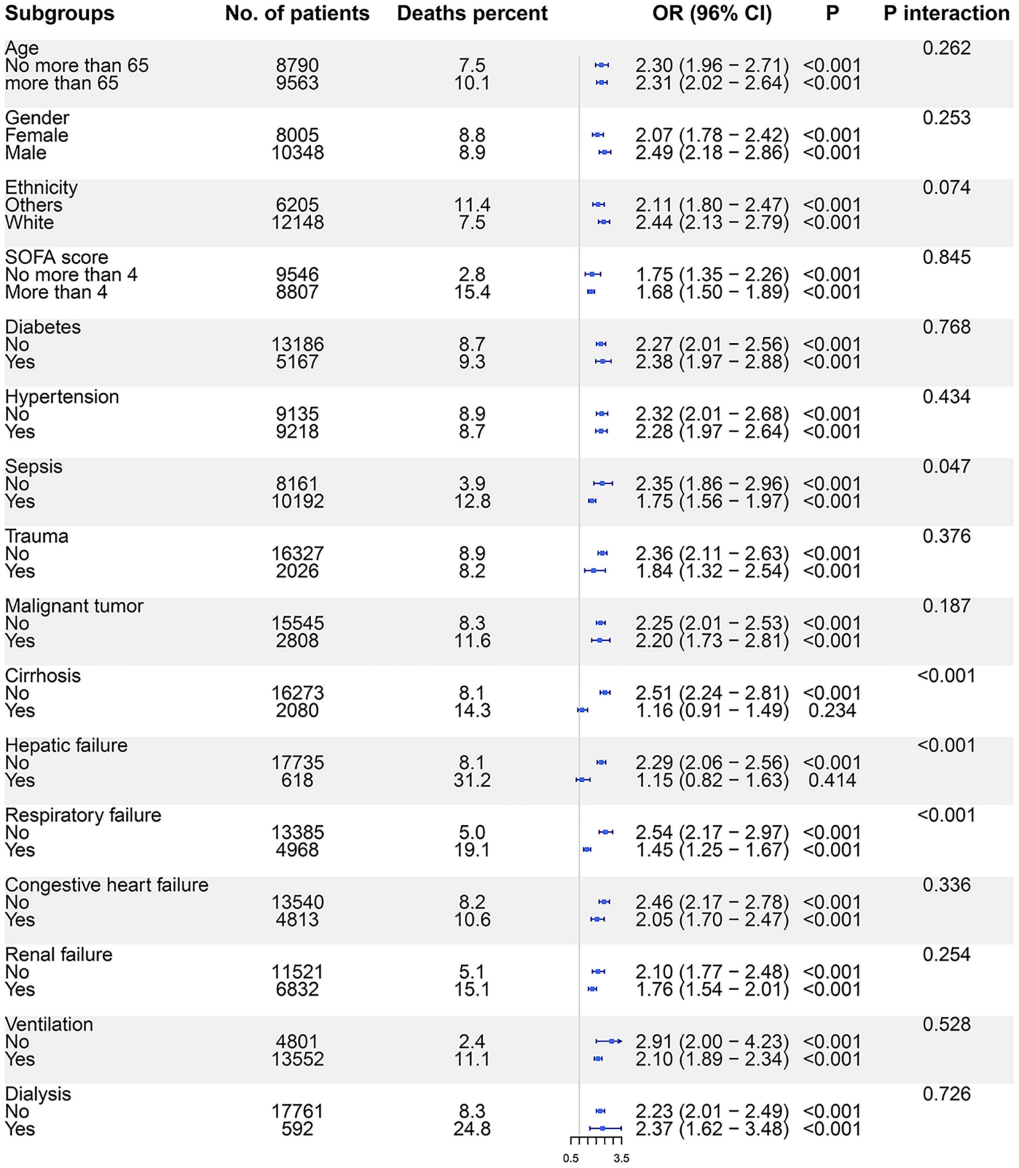
The association between SAL and ICU mortality risks in subgroups. Logistic regression models were used to evaluate the ICU mortality risks for patients with SAL <30 g/l compared with those with SAL ≥30 g/l. SAL, serum albumin level; ICU, intensive care unit; OR, odds ratio; CI, confidence interval.

Based on interaction analyses, we found that the association between SAL and ICU mortality was impacted by the diagnosis of sepsis, cirrhosis, hepatic failure, and respiratory failure. In subgroup analyses, SAL <30 g/l was not associated with ICU mortality in patients with cirrhosis (OR: 1.16, 95% CI: 0.91–1.49, *p* = 0.234) and hepatic failure (OR: 1.15, 95% CI: 0.82–1.63, *p* = 0.414) (Figure 3). The monotonic association between SAL and ICU mortality risk was not observed on the RCS curves plotted in cirrhosis or hepatic failure subgroups (Supplementary Figure S4). The association between SAL and hospital mortality was impacted by gender, SOFA score, diagnosis of sepsis, cirrhosis, hepatic failure, respiratory failure, and renal failure, as well as by the use of ventilation and dialysis (Supplementary Figure S5). In cirrhosis or hepatic failure subgroups, SAL was not monotonically associated with hospital mortality risk either (Supplementary Figure S6).

Among 2,080 patients with cirrhosis, the model for end-stage liver disease-sodium (MELD-Na) score was further calculated (4). In predicting ICU mortality, AUC values (95% CI) for albumin, MELD-Na score, and SOFA score were 0.538 (0.500–0.576), 0.771 (0.742–0.800), and 0.814 (0.786–0.842), respectively. In predicting hospital mortality, AUC values (95% CI) were 0.540 (0.510–0.570), 0.767 (0.744–0.790), and 0.763 (0.740–0.787), respectively (Supplementary Figure S7).

### Validation Cohort

The validation cohort included 63,718 patients from the eICU-CRD database, and its baseline characteristics are presented in Supplementary Table S1. RCS revealed an increasing tendency of ICU mortality as SAL decreased (Supplementary Figure S8). SAL <30 g/l was associated with higher ICU and hospital mortalities (OR: 1.43, 95% CI: 1.33–1.54, *p* < 0.001; OR: 1.62, 95% CI: 1.52–1.71, *p* < 0.001; respectively) even after multivariable adjustment (Table 2). In predicting ICU mortality, AUC values (95% CI) for SAL and APACHE-IV score were 0.690 (0.682–0.697) and 0.856 (0.850–0.862), respectively. In predicting hospital mortality, AUC values (95% CI) for SAL and APACHE-IV score were 0.696 (0.689–0.702) and 0.834 (0.829–0.840), respectively (Supplementary Figure S9).

## Discussion

In this retrospective cohort study with a large number of ICU patients, we found an inverse association of admission SAL with ICU and hospital mortalities. The data from the MIMIC-IV and the eICU-CRD databases both demonstrated that decreased SAL was associated with increased mortality risks, even after multivariable adjustments. However, the association between admission SAL and mortality was affected by the existence of several comorbidities, and it even vanished in patients with cirrhosis or hepatic failure. Besides, the admission SAL had low sensitivity and specificity in outcome prediction.

Low SAL has been considered as a prognostic indicator among patients with a wide range of conditions, including cardiovascular diseases, cirrhosis, sepsis, cancer, and chronic obstructive pulmonary disease (5–12). However, studies on the prognostic value of SAL in general ICUs have been relatively rare and limited in sample size (5, 13–17). A total of 348 critically ill patients were recruited in the study by McCluaky et al. and 1,003 patients were recruited in the study by Yap et al. (13), while 577 patients were recruited in the study by Kendall et al. (5), and 116 patients were recruited in the study by Yin et al. (17). Far surpassing the sample size of the previous studies, our study investigated the prognostic value of admission SAL with 18,353 patients from the MIMIC-IV database and 63,718 patients from the eICU-CRD database. With a large sample size, the present study further demonstrated that decreased SAL at admission was associated with poor outcomes in critically ill patients.

Serum albumin level of 30 g/l has often been considered as the threshold for hypoalbuminemia and the treatment target in clinical trials that aimed to investigate the effect of albumin administration on the prognosis of critically ill patients (2, 18, 19). ROC analysis in the present study also located the optimal cut-off value of SAL at around 30 g/l. Besides, in the MIMIC-IV database, RCS curves revealed rapid increasing risks in ICU and hospital mortalities when SAL declined to below 30 g/l. Therefore, the prognostic value of SAL <30 or ≥30 g/l, as a categorized variable, was further evaluated. Compared with SAL ≥30 g/l, SAL <30 g/l was consistently associated with higher mortality risks in MIMIC-IV and eICU-CRD databases. Therefore, for ICU patients, SAL <30 g/l may serve as a threshold of hypoalbuminemia in clinical practice, which indicated a higher risk of mortality in critically ill patients.

The increased mortality in patients with SAL <30g/l may be explained by the relationship between hypoalbuminemia and underlying poor nutritional status and/or inflammatory process (20–23). However, SAL at admission poorly differentiated survivors and non-survivors in the present study. The SAL had a significantly lower AUC value than the SOFA score or APACHE IV score, presenting low sensitivity and specificity in outcome prediction, which is consistent with previous findings (13, 14, 17). Furthermore, the association between SAL and mortality risk was affected by the existence of comorbidities and even vanished in patients with cirrhosis or hepatic failure. Cirrhotic patients often had a pre-existing inflammatory condition, a decreased synthetic capacity of liver, and an increased distribution volume of albumin due to ascites (24). Recent studies found compromised quality and impaired function of albumin in patients with liver diseases (25). Therefore, the serum albumin concentration may have already been diminished by the chronic conditions, and may be further impacted by the newly emerging deterioration. Besides, even in patients without chronic diseases, serum albumin level may be impacted by acute illness or trauma per se, as well as iatrogenic factors (including fluid resuscitation and albumin infusion). Therefore, a single measurement of SAL at ICU admission may not be able to reliably reflect the clinical state, and may be only a weak prognostic factor in critically ill patients.

Meanwhile, changes in SAL may better reflect the prognosis of patients. Inflammation increases capillary permeability and the escape of serum albumin into interstitial space, and thus decreases the concentration of albumin in serum. Moreover, serving as an essential scavenger and antioxidant, albumin is prone to transfer into bound state during inflammation, and thus has an accelerated degradation rate (20, 26–31). Therefore, decreased SAL is associated with severe inflammation, indicating adverse outcomes, while a rise of SAL is reported to be associated with subsided inflammatory activity and clinical improvement. A study by McClusky et al. (14) observed a more rapid decrease trend of serum albumin during the first 72 h of ICU stay in non-survivors. Kendall et al. (5) found a sharper downward trend of serum albumin in sepsis patients who expired during hospital stay. However, evidence on the association between changes in SAL and clinical outcomes remains sparse, and further studies are warranted to clarify the prognostic value of follow-up albumin levels in critically ill patients.

Several limitations were unavoidable in the present study. First, as mentioned before, SAL in this study was only measured at ICU admission. In the future, our prospective study will adopt follow-up albumin levels, and further investigate the association between the trend of SAL and the improvement or deterioration of clinical state. Second, limited by the retrospective study design, unmeasured potential confounding factors may still remain. Future well-designed and adequately powered, controlled, prospective studies are needed to comprehensively ascertain the presence of underlying confounding factors. Notwithstanding these limitations, our study fills an important gap in the existing literature on the association between SALs and all-cause mortality with a large sample of critically ill patients from ICU databases. The MIMIC-IV and eICU-CRD databases, for instance, with information on trends in vital signs and laboratory measurements of critically ill patients, were used to help machine learning algorithms that provide predictive models for early identification of at-risk patients for improving patient outcomes (32). The extensive use of large databases shows their increasing importance in clinical research (33–36).

In conclusion, ICU patients with SAL <30 g/l had higher ICU and hospital all-cause mortalities than those with SAL ≥30 g/l. However, SAL at admission possesses had low sensitivity and specificity in outcome prediction, especially in patients with cirrhosis. A serial measurement of SAL may better indicate the improvement or deterioration of clinical state of critically ill patients, and should be further clarified in future studies.

## Data Availability Statement

The datasets presented in this study can be found in online repositories. The names of the repository/repositories and accession number(s) can be found below: https://physionet.org/content/mimiciv/1.0/; https://physionet.org/content/eicu-crd/.

## Author Contributions

GW, XJ, DC, and JML designed the study. XJ, LS, JML, JZ, YG, RL, YH, DS, JL, DC, and JR extracted, collected, and analyzed the data. XJ, JZ, and JR prepared the tables and figures. XJ, LS, JL, XW, CW, and GW reviewed the results, interpreted data, and wrote the manuscript. DC, CW, and GW supervised the study. All authors have made an intellectual contribution to the manuscript and approved the submission.

## Funding

Prof. GW received funding from the National Natural Science Foundation of China (81770057).

## Conflict of Interest

CW has received fees for lectures and/or consulting from Daiichi Sankyo, CSL Behring, and Grifols. The remaining authors declare that the research was conducted in the absence of any commercial or financial relationships that could be construed as a potential conflict of interest.

## Publisher's Note

All claims expressed in this article are solely those of the authors and do not necessarily represent those of their affiliated organizations, or those of the publisher, the editors and the reviewers. Any product that may be evaluated in this article, or claim that may be made by its manufacturer, is not guaranteed or endorsed by the publisher.
