# Supplementary material for: Prognostic Value of Serum Albumin Level in Critically Ill Patients: Observational Data From Large Intensive Care Unit Databases
Source: Front Nutr. 2022 Jun 13;9:770674. doi: 10.3389/fnut.2022.770674 (PMC9234460; doi:10.3389/fnut.2022.770674)
Supplement: Supplementary file 1 [file Data_Sheet_1.docx]

Supplementary Material

# Supplementary Figures and Tables

## Supplementary Figures

### Supplementary Figure 1


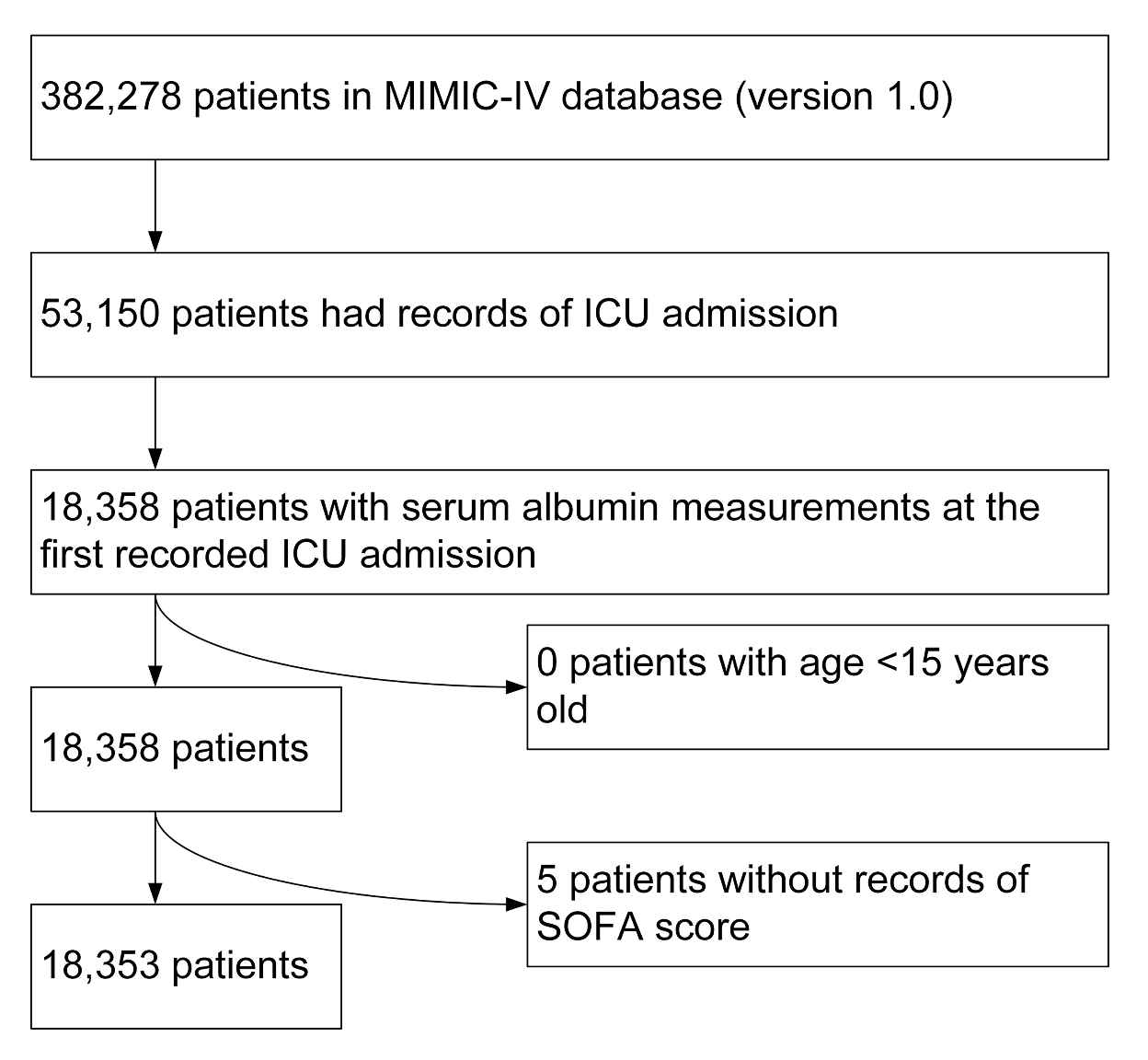


**Supplementary Figure 1.** **Flow chart of participant selection in MIMIC-IV database.** MIMIC-IV, Medical Information Mart for Intensive Care IV; ICU, intensive care unit; SOFA score, Sequential Organ Failure Assessment score.

### Supplementary Figure 2


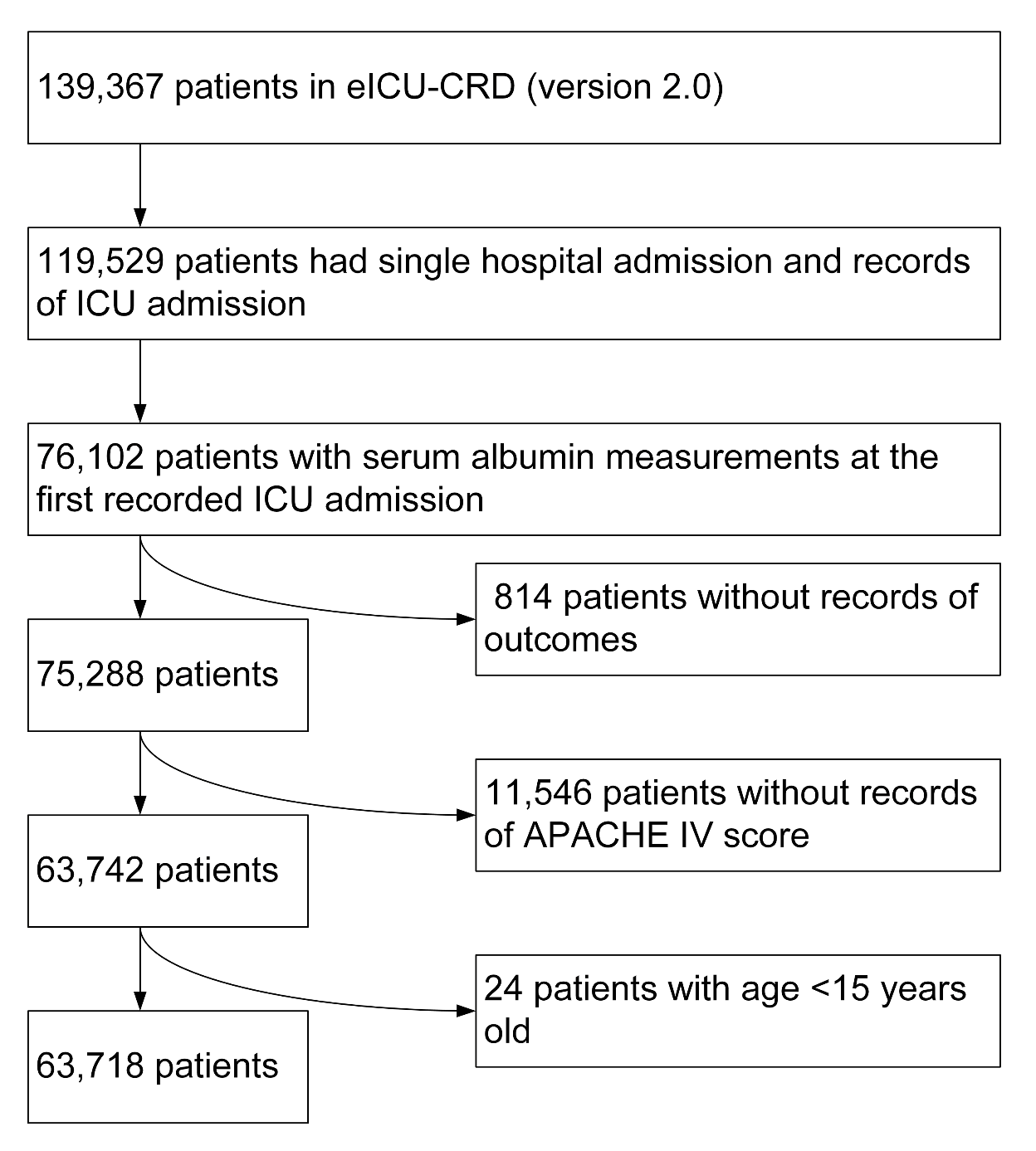


**Supplementary Figure 2.** **Flow chart of participant selection in eICU-CRD.** eICU-CRD, eICU Collaborative Research Database; ICU, intensive care unit; APACHE IV, Acute Physiology and Chronic Health Evaluation IV.

### Supplementary Figure 3


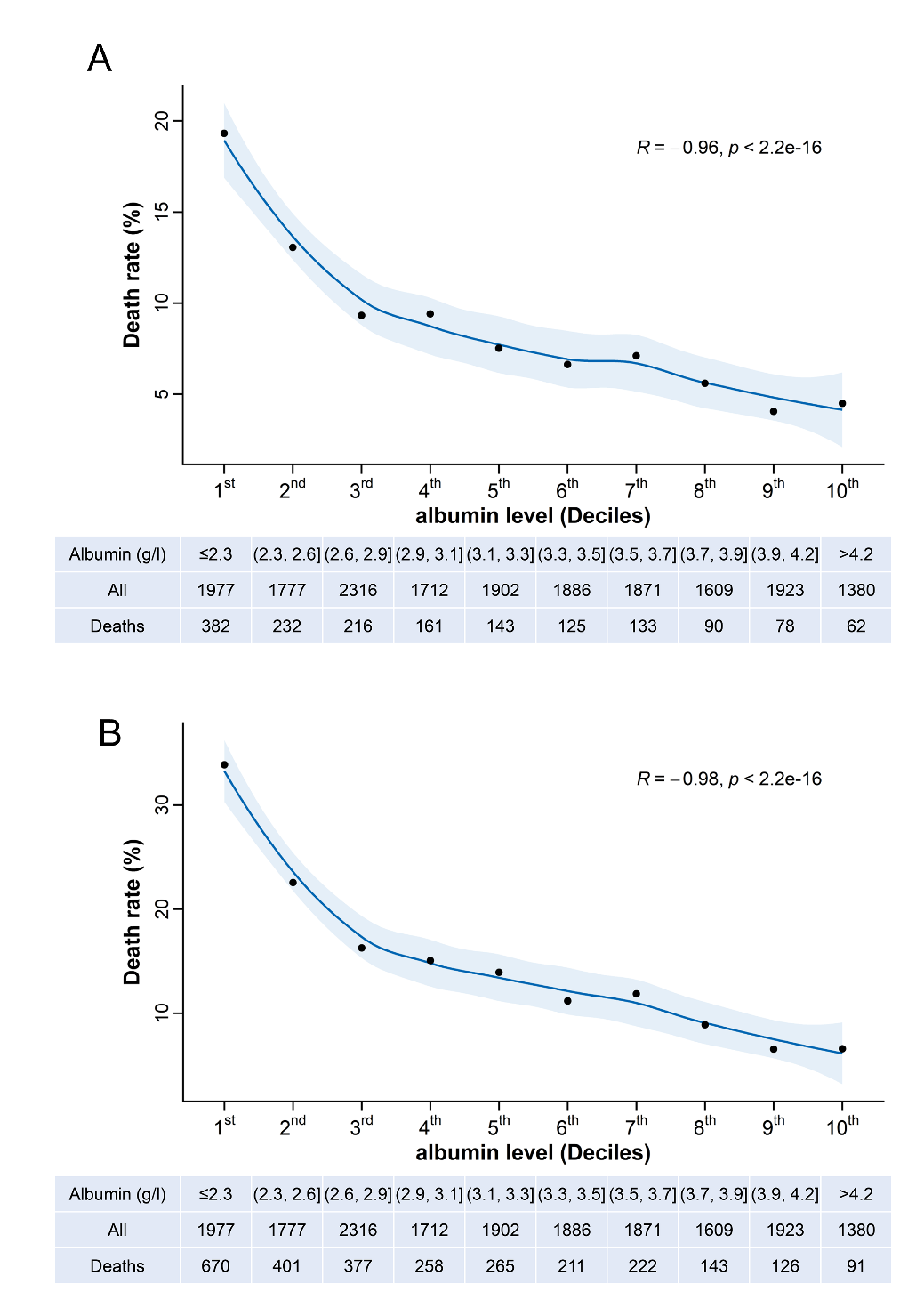


**Supplementary Figure 3.** **The ICU (A) and hospital (B) mortality rates in serum albumin level deciles.** The correlations were measured by Spearman rank sum correlation tests and visualized by Locally Weighted Scatterplot Smoothing curves. ICU, intensive care unit.

### Supplementary Figure 4


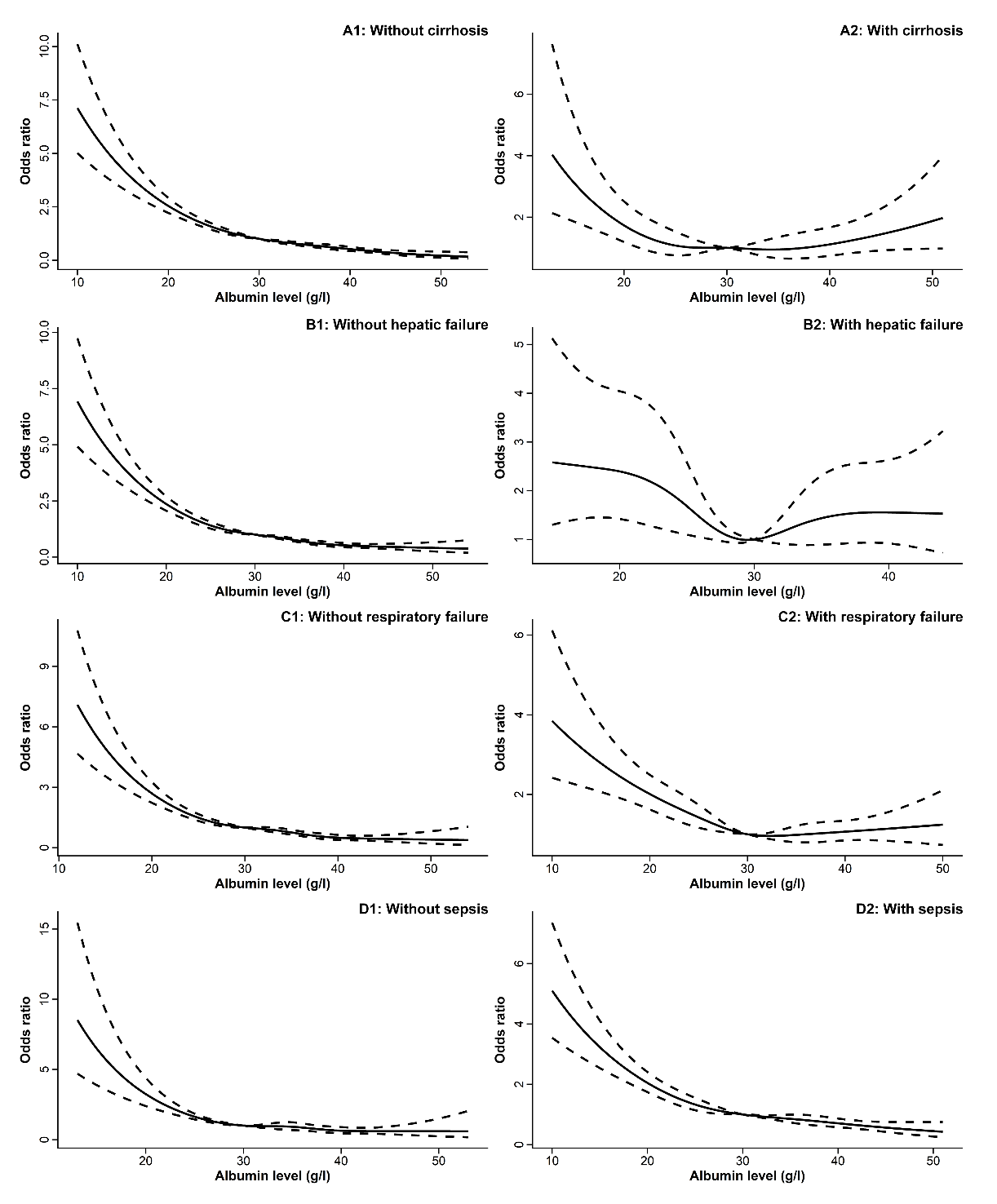


**Supplementary Figure 4.** **The relationships between serum albumin level and the risk of ICU mortalities assessed by restricted cubic splines in subgroups.** Restricted cubic splines were conducted in subgroups with or without cirrhosis, hepatic failure, respiratory failure, and sepsis. ICU, intensive care unit.

### Supplementary Figure 5


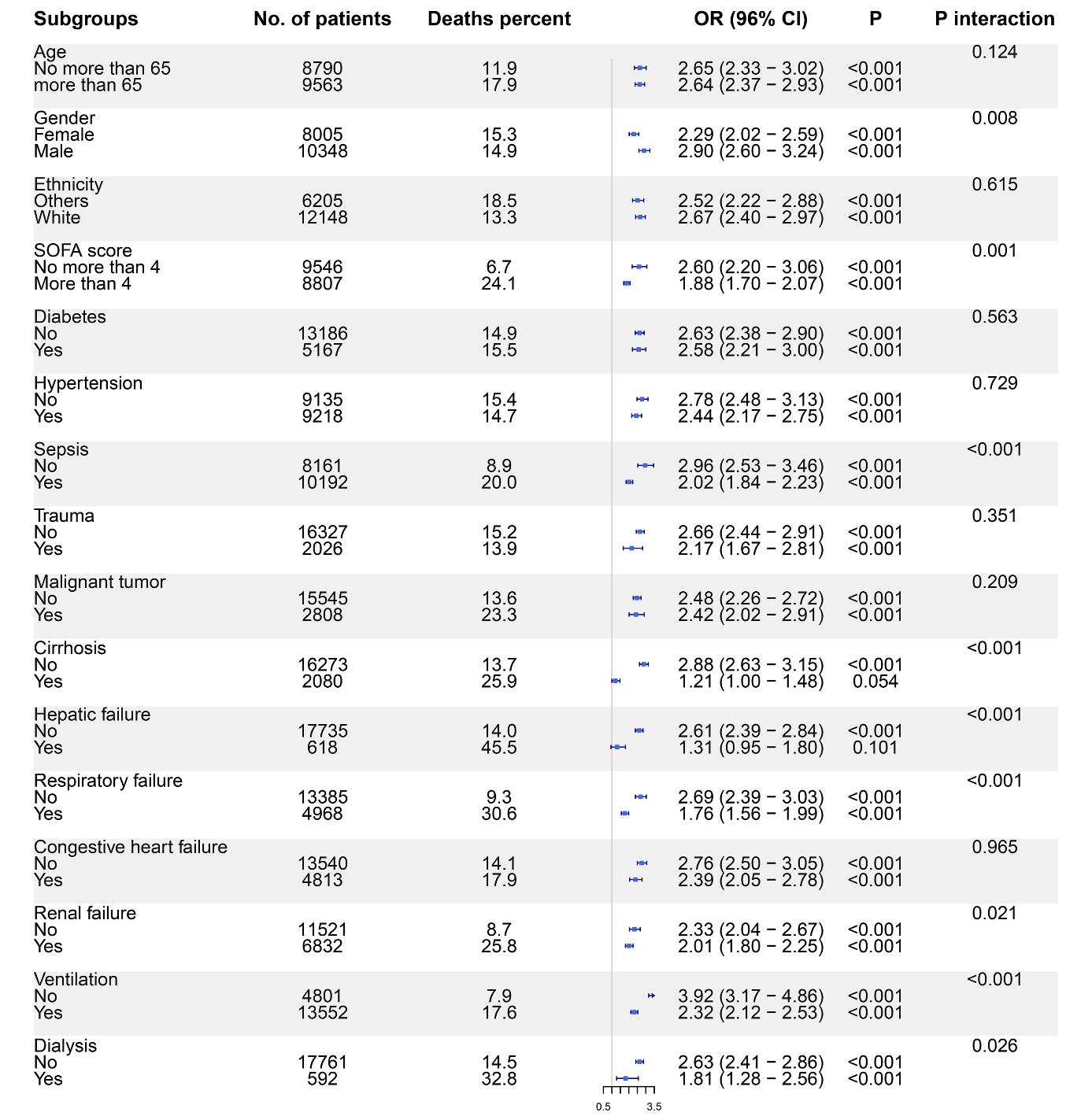


**Supplementary Figure 5.** **The association between serum albumin level and hospital mortality risks in subgroups.** Logistic regression models were used to evaluate the ICU mortality risks for patients with serum albumin level <30 g/L compared with those with serum albumin level ≥30 g/L. ICU, intensive care unit; OR, odds ratio; CI, confidence interval.

### Supplementary Figure 6


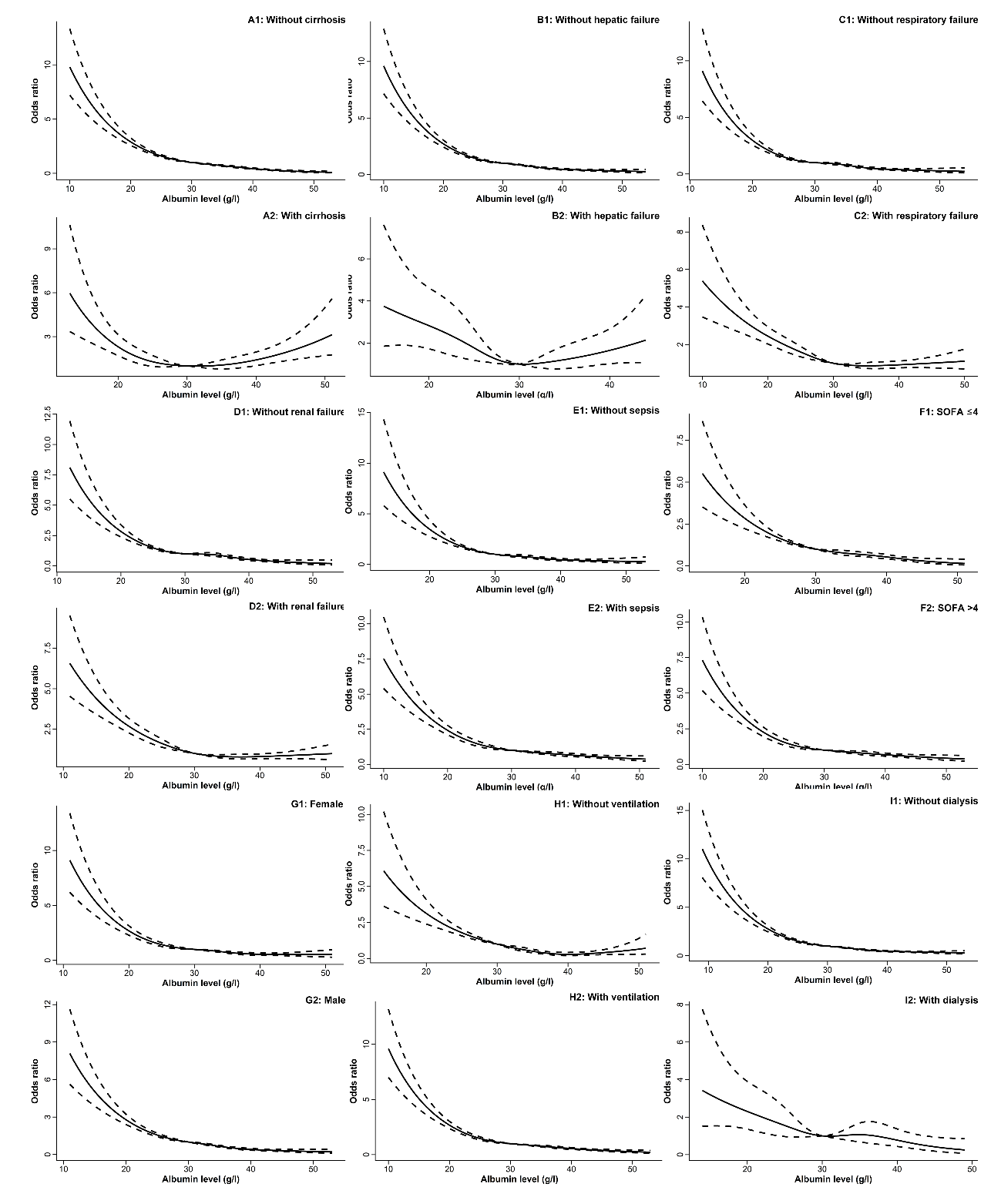


**Supplementary Figure 6.** **The relationships between serum albumin level and the risk of hospital mortalities assessed by restricted cubic splines in subgroups.** Restricted cubic splines were conducted in subgroups with or without cirrhosis, hepatic failure, respiratory failure, renal failure, sepsis, ventilation and dialysis, in female or male, in patients with SOFA score ≤4 or >4. ICU, intensive care unit; SOFA score, Sequential Organ Failure Assessment score.

###
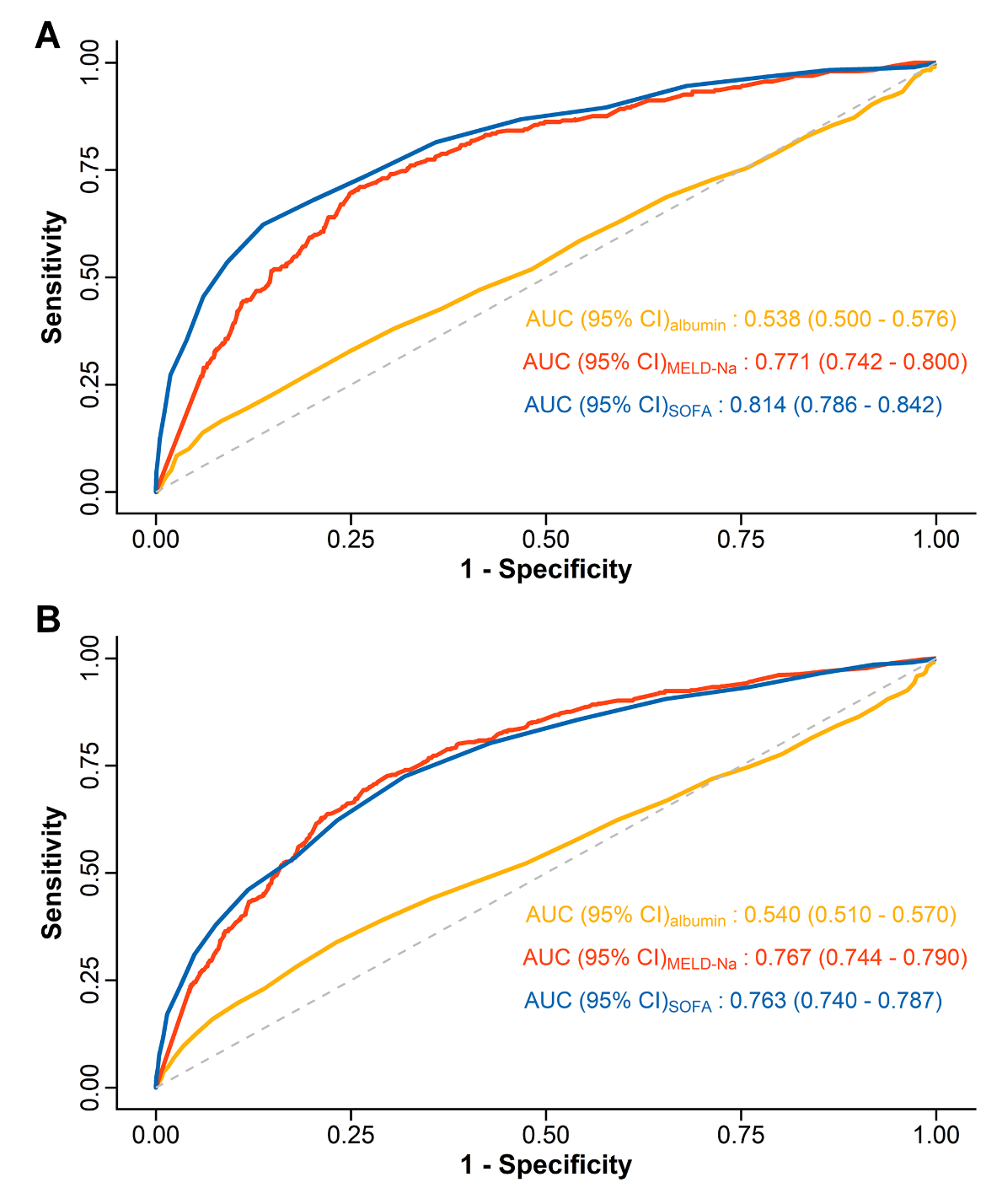
Supplementary Figure 7

**Supplementary Figure 7.** **ROC curves and AUC values for admission serum albumin level, MELD-Na score, and SOFA score in predicting ICU (A) and hospital mortality (B)** **in patients with cirrhosis.** ROC, Receiver operating characteristic; AUC, areas under curve; MELD-Na, the Model for End-stage Liver Disease sodium; SOFA score, Sequential Organ Failure Assessment score; ICU, intensive care unit.

###
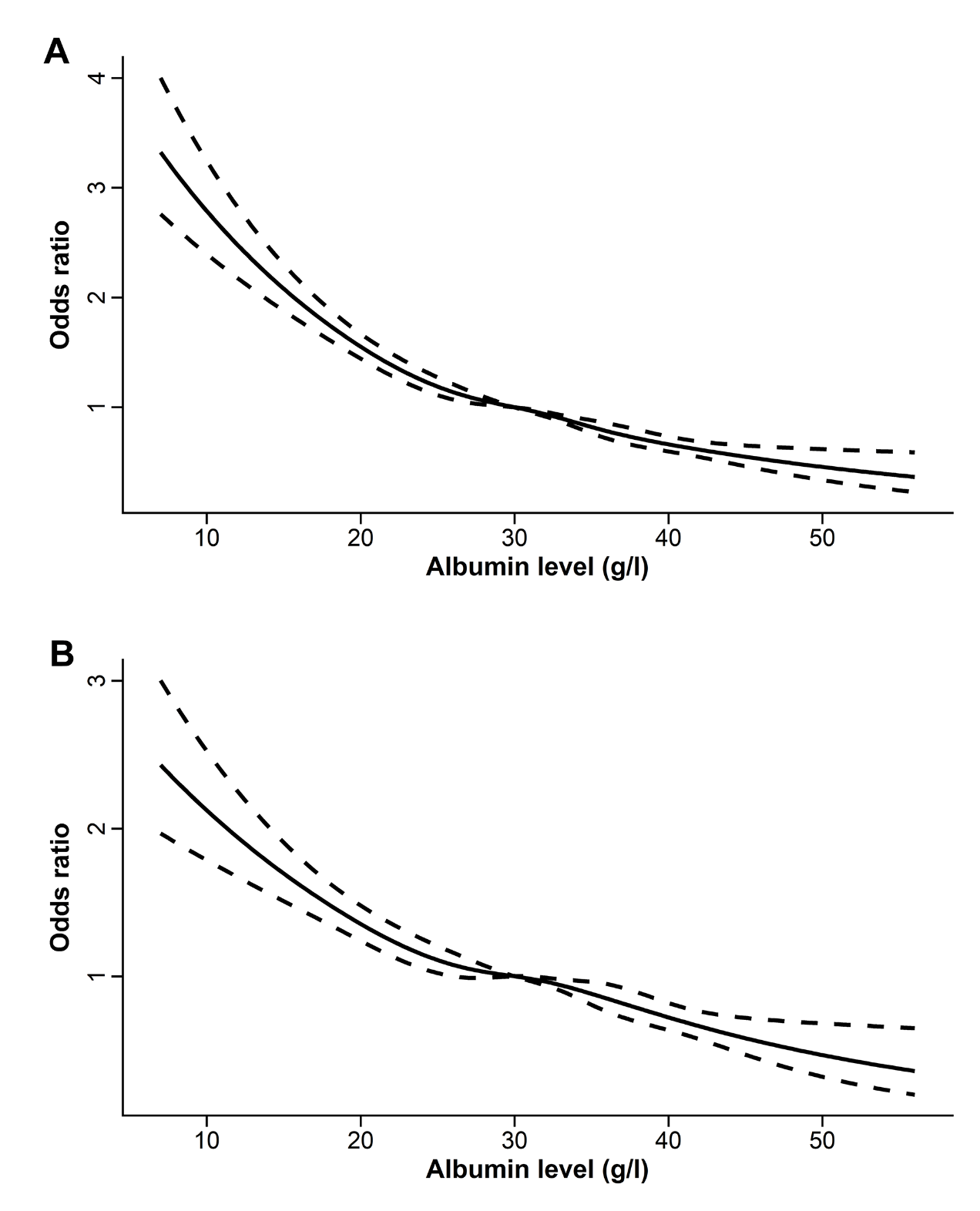
Supplementary Figure 8

**Supplementary Figure 8. The relationships between serum albumin level and the odds ratio of ICU (A) and hospital mortalities (B) assessed by restricted cubic splines in eICU Collaborative Research Database.** ICU, intensive care unit.

###
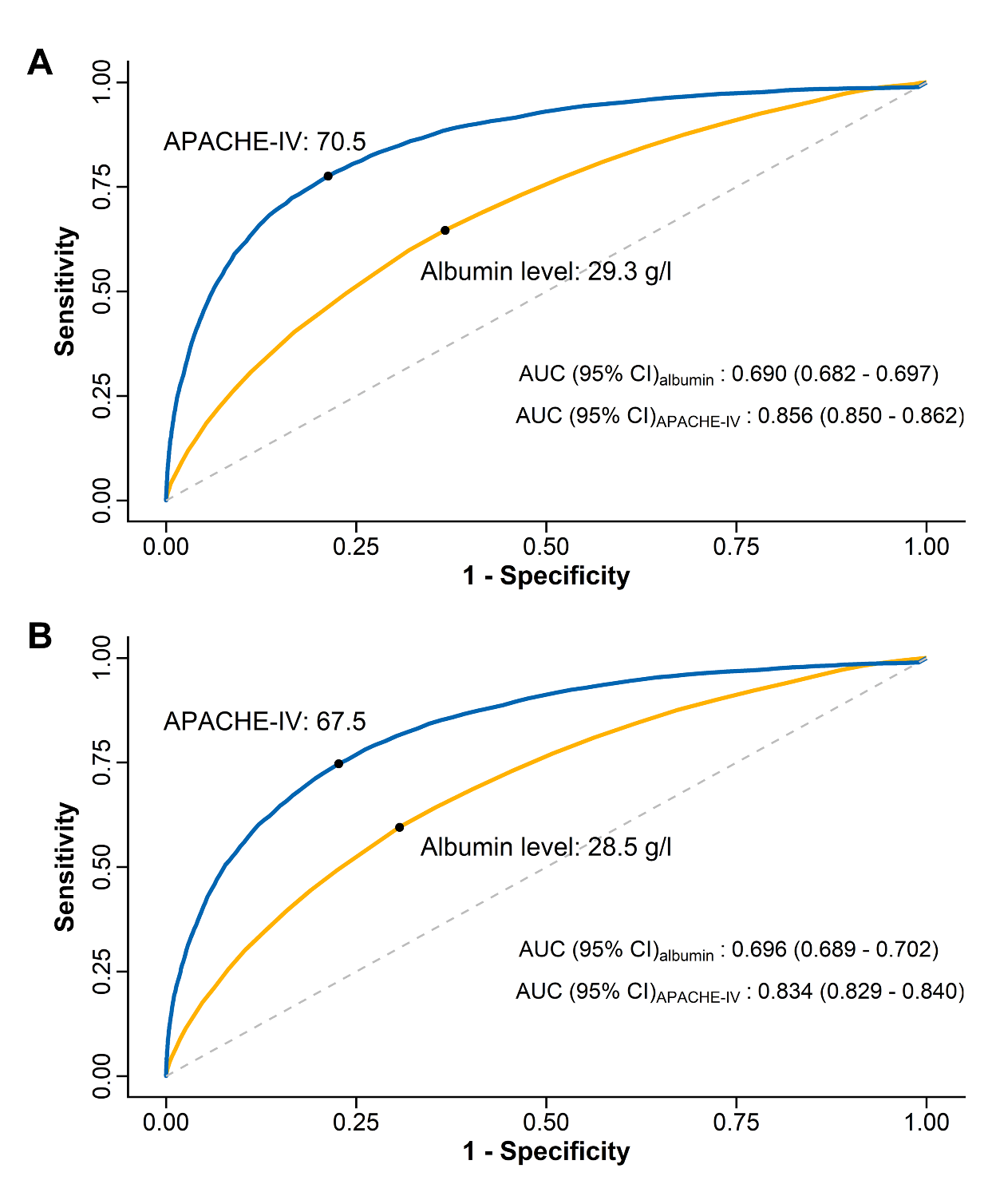
Supplementary Figure 9

**Supplementary Figure 9. ROC curves and AUC values for admission serum albumin level, APACHE IV score in predicting ICU (A) and hospital mortality (B) in eICU Collaborative Research Database.** ROC, Receiver operating characteristic; AUC, areas under curve; ICU, intensive care unit; APACHE IV, Acute Physiology and Chronic Health Evaluation IV.

## Supplementary Table

### Supplementary Table 1

|  | **All**  **(N = 63,718)** | **Albumin <30 g/l**  **(N = 24,732)** | **Albumin ≥30 g/l**  **(N = 38,986)** | **P** |
| --- | --- | --- | --- | --- |
| **Albumin level, Median (IQR), g/l** | 32 (26 - 37) | 25 (21 – 27) | 35 (33 – 39) | <0.001 |
| **Age > 65 years, n (%)** | 30,582 (48.0) | 13,273 (53.7) | 17,309 (44.4) | <0.001 |
| **Gender, n (%)** |  |  |  |  |
| **Male** | 34,498 (54.1) | 12,669 (51.2) | 21,829 (56.0) | <0.001 |
| **Female** | 29,202 (45.8) | 12,059 (48.8) | 17,143 (44.0) |  |
| **Others/Unknown** | 18 (0) | 4 (0) | 14 (0) |  |
| **Ethnicity, n (%)** |  |  |  | 0.127 |
| **White** | 48,742 (76.5) | 18,839 (76.2) | 29,903 (76.7) |  |
| **Others** | 14,976 (23.5) | 5,893 (23.8) | 9,083 (23.3) |  |
| **APACHE score, Median (IQR)** | 52 (37 - 71) | 64 (48 – 83) | 46 (33 – 62) | <0.001 |
| **Comorbidity, n (%)** |  |  |  |  |
| **Diabetes** | 17,348 (27.2) | 7,174 (29) | 10,174 (26.1) | <0.001 |
| **Hypertension** | 31,501 (49.4) | 12,116 (49) | 19,385 (49.7) | 0.072 |
| **Malignant tumor** | 7,691 (12.1) | 3,991 (16.1) | 3,700 (9.5) | <0.001 |
| **Sepsis** | 9,872 (15.5) | 6,676 (27.0) | 3,196 (8.2) | <0.001 |
| **Trauma** | 3,134 (4.9) | 806 (3.3) | 2,328 (6.0) | <0.001 |
| **Cirrhosis** | 1,931 (3.0) | 1,348 (5.5) | 583 (1.5) | <0.001 |
| **Hepatic failure** | 180 (0.3) | 129 (0.5) | 51 (0.1) | <0.001 |
| **Congestive heart failure** | 1,972 (3.1) | 689 (2.8) | 1,283 (3.3) | <0.001 |
| **Respiratory failure** | 598 (0.9) | 277 (1.1) | 321 (0.8) | <0.001 |
| **Renal failure** | 1,005 (1.6) | 458 (1.9) | 547 (1.4) | <0.001 |
| **Treatments, n (%)** |  |  |  |  |
| **Ventilation** | 15,935 (25.0) | 7,561 (30.6) | 8,374 (21.5) | <0.001 |
| **Dialysis** | 2,686 (4.2) | 1,533 (6.2) | 1,153 (3.0) | <0.001 |
| **Outcomes, n (%)** |  |  |  |  |
| **Hospital death** | 7,493 (11.8) | 4,814 (19.5) | 2,679 (6.9) | <0.001 |
| **ICU death** | 4,887 (7.7) | 3,159 (12.8) | 1,728 (4.4) | <0.001 |

**Supplementary Table 1. Demographic and clinical characteristics at baseline for patients from eICU Collaborative Research Database.** APACHE score, acute physiology and chronic health evaluation IV score; IQR, inter-quartile range; ICU, intensive care unit.
